# The Single-Breath Diffusing Capacity of CO and NO in Healthy Children of European Descent

**DOI:** 10.1371/journal.pone.0113177

**Published:** 2014-12-16

**Authors:** Astrid Thomas, Birgitte Hanel, Jacob L. Marott, Frederik Buchvald, Jann Mortensen, Kim G. Nielsen

**Affiliations:** 1 Danish PCD & chILD Centre, CF Centre Copenhagen, Pediatric Pulmonary Service, Department of Pediatrics and Adolescent Medicine, Rigshospitalet, University of Copenhagen, Copenhagen, Denmark; 2 The Copenhagen City Heart Study, Frederiksberg Hospital, Copenhagen, Denmark; 3 Department of Clinical Physiology, Nuclear Medicine and PET, Rigshospitalet, University of Copenhagen, Copenhagen, Denmark; Catholic University of the Sacred Heart, Italy

## Abstract

**Rationale:**

The diffusing capacity (D_L_) of the lung can be divided into two components: the diffusing capacity of the alveolar membrane (Dm) and the pulmonary capillary volume (Vc). D_L_ is traditionally measured using a single-breath method, involving inhalation of carbon monoxide, and a breath hold of 8–10 seconds (D_L,CO_). This method does not easily allow calculation of Dm and Vc. An alternative single-breath method (D_L,CO,NO_), involving simultaneous inhalation of carbon monoxide and nitric oxide, and traditionally a shorter breath hold, allows calculation of Dm and Vc and the D_L,NO_/D_L,CO_ ratio in a single respiratory maneuver. The clinical utility of Dm, Vc, and D_L,NO_/D_L,CO_ in the pediatric age range is currently unknown but also restricted by lack of reference values.

**Objectives:**

The aim of this study was to establish reference ranges for the outcomes of D_L,CO,NO_ with a 5 second breath hold, including the calculated outcomes Dm, Vc, and the D_L,NO_/D_L,CO_ ratio, as well as to establish reference values for the outcomes of the traditional D_L,CO_ method, with a 10 second breath hold in children.

**Methods:**

D_L,CO,NO_ and D_L,CO_ were measured in healthy children, of European descent, aged 5–17 years using a Jaeger Masterscreen PFT. The data were analyzed using the Generalized Additive Models for Location Scale and Shape (GAMLSS) statistical method.

**Measurements and Main Results:**

A total of 326 children were eligible for diffusing capacity measurements, resulting in 312 measurements of D_L,CO,NO_ and 297 of D_L,CO_, respectively. Reference equations were established for the outcomes of D_L,CO,NO_ and D_L,CO_, including the calculated values: Vc, Dm, and the D_L,NO_/D_L,CO_ ratio.

**Conclusion:**

These reference values are based on the largest sample of children to date and may provide a basis for future studies of their clinical utility in differentiating between alterations in the pulmonary circulation and changes in the alveolar membrane in pediatric patients.

## Introduction

The transfer factor of the lung for a gas, is often called the diffusing capacity of the lung (D_L_). D_L_ for an inhaled gas reactive with hemoglobin is the flow of that gas from the alveoli to the blood for a unit difference in pressure. D_L_ can be divided into two components: the diffusing capacity of the pulmonary membrane (Dm) and the chemical reaction of the gas binding to the blood. The latter is determined by the specific conductance of blood for a given gas, *Θ*, and the capillary volume of the lung (Vc).

The single-breath method was first introduced in 1915 [1]. Today, the single-breath D_L_ of carbon monoxide (CO) using a breath-hold of 10 seconds (D_L,CO,10s_) is the most frequently used method with the current ATS/ERS methodological guidelines [2].

In 1957, Roughton and Forster proposed a method of calculating Dm and Vc, using D_L,CO,10s_, which required arterial samples and two respiratory maneuvers at two different oxygen tensions [3]. In 1987, Guénard, Varène and Vaida [4] proposed an alternative method (D_L,CO,NO_) of determining Vc and Dm involving simultaneous inhalation of CO and nitric oxide (NO). Both CO and NO transfer are diffusion limited, but NO has approximately twice the physical diffusivity of CO, and the affinity to hemoglobin for NO (*Θ*_NO_) is approximately 250 times greater [5]. The implications have been described in detail elsewhere, but in summary *Θ*_NO_ was previously assumed infinitely great [4]. However, recent studies have challenged this assumption, leading to proposal of a finite value of *Θ*_NO_. The consequence of the use of a finite value for NO blood conductance is that D_L,NO_ appears equally dependent on Dm and Vc as D_L,CO_ is mainly dependent on Vc. [6], [7].

The calculation of Dm and Vc involves the resistance of the red blood cell to gas transfer (*Θ*_gas_), but no consensus currently exists about the true value of *Θ*_CO_.

With the previous assumption of an infinite value of *Θ*_NO_, calculation of the D_L,NO_/D_L,CO_ ratio was thought to provide useful information about the differentiation between primary alveolar membrane impairment (low D_L,NO_/D_L,CO_ ratio) [8], [9], [10] or abnormalities of the pulmonary circulation (high D_L,NO_/D_L,CO_ ratio) [11], potentially providing additional insights into more specific factors affecting D_L_ [12]. Now that a finite value of *Θ*_NO_ has been determined, new interpretations of the ratio will be necessary.

Determination of Vc and Dm using D_L,CO,NO_ requires a single respiratory maneuver and allows simultaneous determination of D_L,CO_, D_L,NO_, as well as calculation of D_L,NO_/D_L,CO_, Dm, and Vc. In addition, D_L,CO,NO_ generally involves a shorter breath-hold due to the fast disappearance of NO [4]. The present study used a breath-hold of 5 seconds (D_L,CO,NO,5s_). Reference equations for these outcomes of D_L,CO,NO,5s_ in children are scarce. A study involving 50 children over 8 years of age has been published [13], as well as a more recent study involving 85 healthy North African boys, aged 8–16 years [14] whereas two larger studies recently produced reference equations for the more frequently used outcomes of D_L,CO,10s_ [15], [16].

Despite similarities in the performed respiratory maneuver D_L,CO,NO,5s_ and D_L,CO,10s_ are two distinctly separate methods, with multiple methodological differences.

The primary goal of this study was to calculate reference equations for the outcomes of D_L,CO,NO,5s_ including Dm, Vc, and the D_L,NO_/D_L,CO_ ratio, in healthy children. Since no consensus guidelines exist for D_L,CO,NO,5s_ and previous data is limited, contemporary measurement of the frequently used D_L,CO,10s_ was performed to allow assessment of correlation between these two substantially different techniques and to assess whether they could be used interchangeably, although, knowing for a fact, that significant methodological differences exist. The resulting measurements of D_L,CO,10s_ allowed establishment of reference equations and comparison with existing published reference equations for D_L,CO,10s_. Some of the results of this study have been previously reported in the form of an abstract [17].

## Materials and Methods

The regional ethics committee of Copenhagen (“De Videnskabsetiske Komiteer i Region Hovedstaden”) approved the project, and all subjects and/or their parents provided written, informed consent (approval number: H-4-2011-111).

### Design and Subjects

In this cross-sectional, single-center study, healthy children and adolescents aged between 5 and 17 years were recruited from December 2011 to August 2012 from a private combined elementary and high school in Copenhagen, a public elementary school in rural Denmark, and among the healthy siblings of patients, and the children of staff at the Danish Pediatric Pulmonary Service. Prior to participation, the children (>15 years) or their parents were asked to fill out a health questionnaire covering gestational age, previous or current pulmonary disease, atopic illness, allergies, and any additional diseases the child had had, as well as current and previous medications.

All participants were non-smokers, had two parents of European descent, and had no current pulmonary or cardiac disease, including any upper or lower respiratory infection 2 weeks prior to the measurements. Any use of bronchodilators, and in particular, use in the day previous to participation, was considered an exclusion criterion. Furthermore, we excluded participants with FEV_1_/FVC below the age- and weight-specific lower limit according to recent data [18] or who were unable to co-operate or perform adequate respiratory maneuvers.

### Methods

Height and weight were measured without shoes to the nearest 0.1 cm and 100 grams, respectively, using standard stadiometers (Seca, Hamburg, Germany) and scales. Age was calculated by difference between date of birth and participation date, and was recorded to decimal accuracy.

Hemoglobin concentration was measured by a finger stick blood sample test (The HemoCue Hb 201+; HemoCue, Denmark) in all participants unless the child refused. Correction for hemoglobin concentration is not imperative in healthy children, as variations within the normal range do not significantly affect D_L,CO_ [19]. In children who refused hemoglobin measurement, we assumed normal values of 13.4 g/dL (8.3 mmol/L) for females, as well as males up to 15 years of age, and 14.6 g/dL (9.0 mmol/L) for males >15 years of age according to ATS/ERS guidelines [2].

### Measurements of lung function

Spirometry, D_L,CO,NO,5s_, and D_L,CO,10s_ were performed using the Jaeger Masterscreen PFT pro (CareFusion, Hoechberg, Germany). Two identical sets of equipment were used at the three locations: one was used at the two participating schools and the other at the Danish Pediatric Pulmonary Service. Two experienced technicians performed all of the measurements. For most participants, spirometry and measurements of diffusing capacity were performed in a single sitting, but occasionally it required two sittings due to weariness with decreasing ability to perform technically acceptable measurements, especially with the younger children. If a participant was not able to make technically acceptable measurements in all three pulmonary function tests during the first sitting, they were invited back a second time. Spirometry always preceded the diffusing capacity measurements; D_L,CO,NO,5s_ and D_L,CO,10s_ were performed in a random order except in the youngest children, in whom D_L,CO,NO,5s_ was measured first because it was the primary goal of this study.

Participants breathed through a single-use mouthpiece with a built-in bacterial/viral filter (Spirobach, Tyco, Healthcare, Italy) connected to the pneumotachograph.

### Diffusing capacity measurements

Participants were instructed to breathe normally. Following two to three normal breaths, participants performed a deep expiration and then a complete and fast inspiration. Following a breath-hold, a complete and smooth expiration was performed. As stated in the introduction D_L,CO,NO,5s_ and D_L,CO,10s_ are performed with a identical respiratory maneuver, with the exception of breath-hold time, but it is important to clarify that they are two distinctly separate methods, contained within one equipment setup, with differences in test gasses, gas analyzers and sampling techniques.

See Table 1 for specific methodological differences between D_L,CO,NO,5s_, and D_L,CO,10s_.

**Table 1:** Summary of methodology.

	D_L,CO,NO,5s_	D_L,CO,10s_
**Breath-hold**	5 seconds	10 seconds
**Gas mixture**	0.3% CO, 9% He, 20.9% O_2_, 69.8% N_2_ mixed with 400 ppm NO/O_2_ *	0.3% CO, 0.3% CH_4_, 20.9% O_2_, and balanced N_2_
**Inert gas**	Helium	Methane
**Gas analyzer** †	NO: CiTicel 7BNT electrochemical cell, CO: Electrochemical Cell, He: Thermal Conductivity, O_2_: Electrochemical Cell	CO, CH_4_: Non-dispersive infrared thermopile
**Gas sampling method**	Physical sample from collection bag	Virtual sample constructed from flow and gas concentration signals.
**CO_2_ -correction** ‡	4,5%	-

**D_L,CO,NO,5s_** represents the single-breath diffusing capacity for NO and CO with a 5-second breath-hold. **D_L,CO,10s_** represents the single-breath diffusing capacity for CO with a 10-second breath-hold. Inert gas was used to measure the alveolar volume (V_A_).

*The concentration of NO in inspired gas was approximately 50 PPM according to the standard settings of the equipment.

† City Tech. Ltd produced all gas analyzers.

‡ CO_2_ correction is applied due to cross-sensitivity of the Helium Analyser with CO_2_.

Quality control was performed separately for the two methods. Having unacceptable measurements for one method did not exclude the participant from attempting to perform the other method. The average of two acceptable tests for each method was reported and included in data analysis.

We required at least 4 minutes between each measurement, to allow adequate elimination of the test gases. Discard and sample volume were each 600 ml in both D_L,CO,NO,5s_ and D_L,CO,10s_. For children with a VC <1.5 L we reduced the discard volume to 500 ml [2]. The gas concentration curves were viewed prior to sample collection to confirm that dead space washout was complete.

Breath-holding time was calculated using the Jones and Mead method for both D_L,CO,NO,5s_ and D_L,CO,10s_ [20].

The instrument dead space for both D_L,CO,NO,5s_ and D_L,CO,10s_ (V_D, ins_) was 130 ml, and the anatomical dead space (V_D,an_) was calculated according to Cotes formula from 1993 as V_D, an_ = 2.2 ml/kg 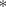 weight in kg [21].

Alveolar volume (V_A_) was calculated using the following formula:
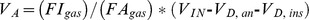
where FI_gas_ is the inspiratory fraction of inert gas (Methane or Helium for D_L,CO,10s_ and D_L,CO,NO,5s_ respectively) and FA_gas_ is the alveolar fraction of inert gas. V_IN_ is the inspiratory volume.

All measurements were performed at sea level. D_L,CO_ and the diffusing capacity for CO per unit of alveolar volume (D_L,CO_/V_A_ = K_CO_) were corrected for hemoglobin concentration when available. D_L,NO_ and the diffusing capacity for NO per unit of V_A_ (D_L,NO_/V_A_ = K_NO_) were not corrected for hemoglobin concentration [6].

D_L,CO,NO5s_ and D_L,CO,10s_ were performed according to current ATS/ERS guidelines [2], though we considered a ratio between inspiratory volume and FVC (V_IN_/FVC) >80% as sufficient, in contrast to a ratio >85%. The vital capacity (VC) was not measured in our subjects, but FVC acquired during spirometry was assumed to be equivalent to the VC, as FVC has been shown to not differ significantly from VC in healthy subjects [22], [23].

Both D_L,CO,10s_ and D_L,CO,NO,5s_ result in the measurement of D_L,CO_, V_A_, and K_CO_. In addition, D_L,CO,NO,5s_ produces measurements of D_L,NO_, K_NO_, and allows calculation of Dm, Vc, and D_L,NO_/D_L,CO(5s)_. To differentiate between the two methods, D_L,CO,10s_ outcomes are denoted with “10s” and D_L,CO,NO,5s_ outcomes with “5s” in this paper, e.g., V_A,10s_ for V_A_ measured using D_L,CO,10s_.

### Quality control of equipment

Volume and gas calibration and biological quality control was performed daily prior to the measurements. Calibration syringes were tested for volume accuracy and were in accordance with ATS/ERS standards [2]. Gas-analyzers were factory checked and quality controlled for linearity as required for the D_L,CO,10s_ method before start of the study and after completion of the study in both sets of equipment, and were found in accordance with ATS/ERS standards. A quality control report on both sets of equipment is provided in Supporting Information. **Appendix S1.** Biological quality control of measurements using both D_L,CO,10s_ and D_L,CO,NO,5s_ in addition to assessments of volumes demonstrated high levels of repeatability within subjects, between session and between equipment setups during the entire study period.

### Calculation of *Θ* and Vc

Roughton and Forsters' 1/*Θ*CO value at pH 7.4 was used [24]:
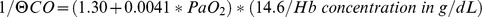
PaO_2_ was set at 100 mmHg. Using the standard hemoglobin concentrations, 1/*Θ*CO was found to be 1.71 for females and for males <15 years of age, and 1.86 for males >15 years of age.

A Dm_NO_/Dm_CO_ ratio (α) of 1.97 [4] was used, and Vc was determined by isolating 1/Vc in the following equation:
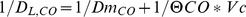
The calculations above assume the previously acknowledged infinite value of *Θ*_NO_. Recently a finite value of *Θ*_NO_ has been accepted as more accurate, and was therefore used in this study. The finite value of *Θ*_NO_ leads to the following equations:
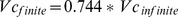
and
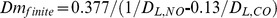

[25]

### Statistical analysis

The primary outcomes for D_L,CO,NO,5s_ were considered to be D_L,CO,5s_, K_CO,5s_, V_A,5s_, D_L,NO_, K_NO_, and the calculated outcomes D_L,NO_/D_L,CO,5s_, and Vc, Dm for the finite value of *Θ*_NO_. Primary outcomes for D_L,CO,10s_ were D_L,CO,10s_, K_CO,10s_, and V_A,10s_. Reference equations were established using Generalized Additive Models for Location Scale and Shape (GAMLSS) with extended capabilities compared to the simpler, generalized linear models. The GAMLSS regression analysis allows the median or mean value (*mu*), the variability (*sigma*), and the skewness (*nu*) of the outcome variable to change with the explanatory variables. Possible distributions for the GAMLSS models were normal distribution (linear regression with *mu* and *sigma*), gamma distribution (*mu* and *sigma*), or the Box-Cox Cole and Green (BCCG) distribution (*mu*, *sigma*, and *nu*). The latter is suitable for skewed data.

Stepwise model selection was carried out using the Generalized Akaike Information Criterion (GAIC). Possible explanatory variables in the selection of *mu*, *sigma*, and *nu* were age, sex, height, and cube of height, as well as any two-way interaction between these variables for mu. Goodness of fit was assessed by ‘worm plots’ and Q statistics [26], [27]. For all three distributions we investigated models with log mu links, log sigma links and for the Box-Cox Cole Green distribution identity nu links. Measurements not meeting ATS quality criteria (>10% difference between to measurements, and V_IN_/FVC between 80% and 85%) were included after evaluating the influence and leverage of the resulting data points in ordinary linear regression analysis [28], [29], [30], [31]. All analyses were performed using the statistical software R (version 3.0.2; R Foundation, http://www.r-project.org) including the GAMLSS package.

## Results

**Figure 1:**
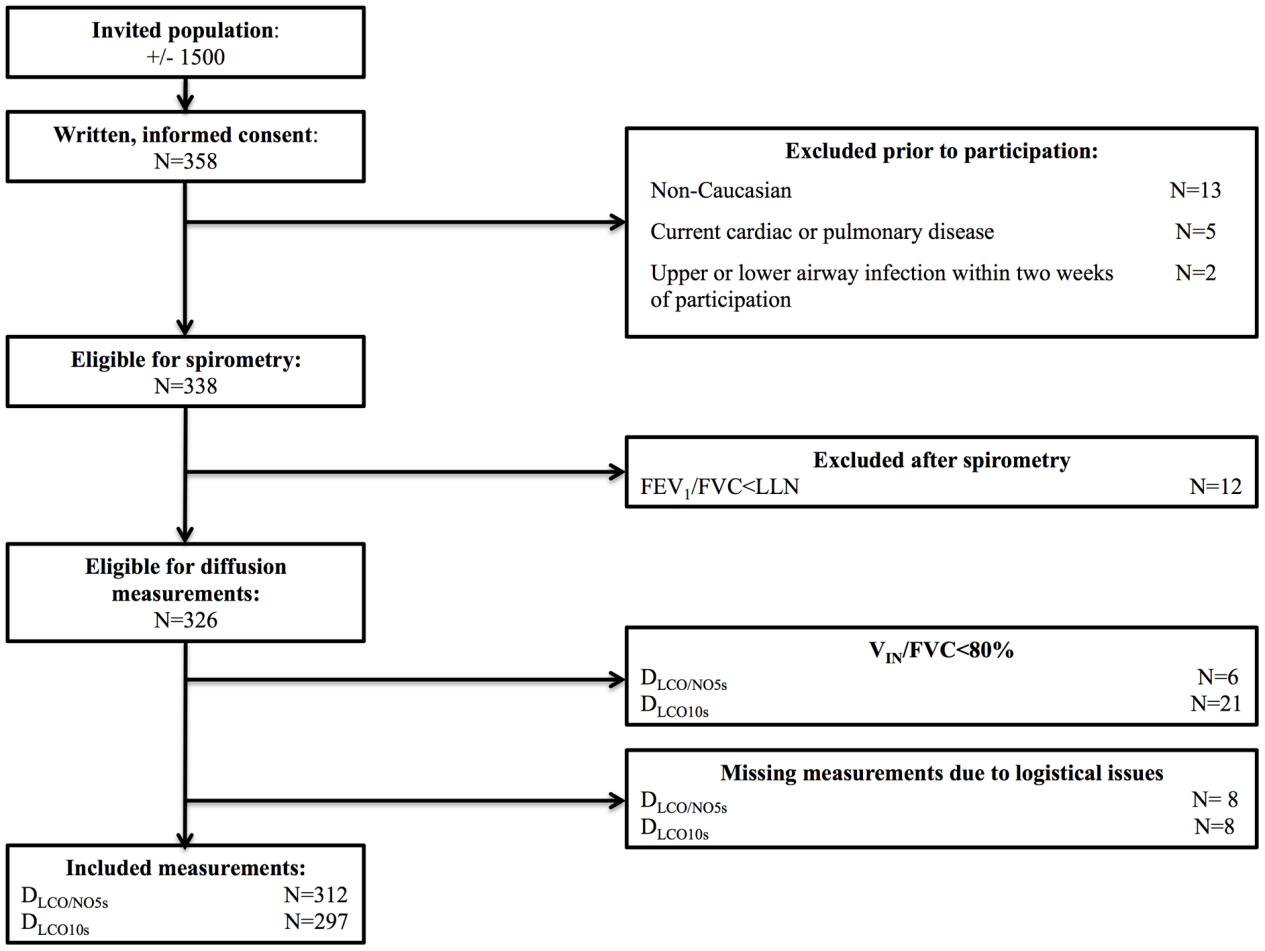
Inclusion flowchart. Invitations to participate were sent to approximately 1500 children, of which 358 participants and/or their parents provided informed consent.

See figure 1 for the inclusion flow chart. Baseline characteristics are provided in table 2. The populations in our three locations were similar in all regards. See **Figure S1** for the age distribution.

**Table 2:** Baseline characteristics at the three locations.

		Private school, Copenhagen	Pediatric Pulmonary Service, Copenhagen	Public school, rural Denmark
N		159	55	112
Sex (male)	N (%)	82 (51.6)	24 (43.6)	55 (49.1)
Age (y)	Mean (SD) [range]	11.4 (3.3) [5–17]	11.5 (4.4) [5–17]	10.3 (2.9) [5–16]
Height (cm)	Mean (SD) [range]	152.7 (19.3) [104.9–187.6]	150.5 (24.2) [107.4–186.8]	145.5 (16.8) [108.0–182.0]
Weight (kg)	Mean (SD) [range]	45.2 (16.3) [18.8–93.6]	45.9 (19.2) [14.8–81.5]	40.1 (14.8) [18.1–101.2]
FEV_1_ (Z-score)	Mean (SD) [range]	1.17 (0.93) [−1.05–3.62]	0.73 (0.83) [−1.29–2.82]	1.14 (0.93) [−1.20–3.38]
FVC (Z-score)	Mean (SD) [range]	1.16 (0.95) [−0.89–3.97]	0.62 (0.81) [−1.01–2.50]	1.11 (1.04) [−1.31–4.10]

FEV_1_ = forced expiratory volume in one second, FVC = forced vital capacity, SD = standard deviation.

**Figure 2:**
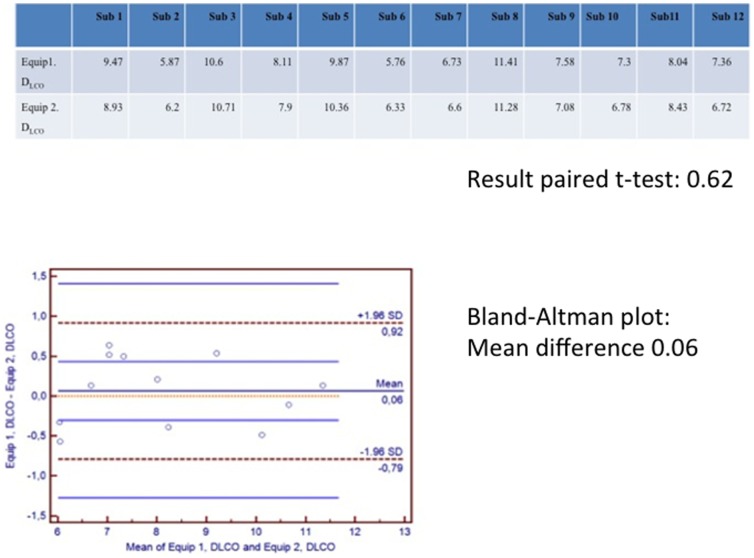
Comparison using Bland and Altman plots of results in 12 subjects assessed by the two sets of equipment used. One at the Pediatric Pulmonary Service, Copenhagen and the other at the two schools involved.

Conformity between the two sets of equipment for D_L,CO,10s_ was evaluated using a paired t-test (p = 0.62) and a Bland-Altman plot (mean difference = 0.06). See figure 2.

### Reference equations

Reference equations, as well as the sigma for all outcomes, are presented in table 3. In addition please see the provided excel spreadsheet, that allows calculation of predicted reference values.

**Table 3:** Reference equations for D_L,CO,NO,5s_ and D_L,CO,10s_.

D_L,CO,NO,5s_	Model	Equation ( = mu)	Coefficient of variation ( = Sigma)
D_L,NO_, (mmol/min)/kPa*	Gamma	exp(1.3145+0.0214*A−0.0058*S+0.0119*H−1.2893*10^∧^−8 *H^∧^3+2.7070*10^∧^−8*S*H^∧^3)	exp(−2.2490)
K_NO_, ((mmol/min)/kPa)/L‡	Normal	exp(1.2672+1.1168*S+0.0098*H−1.8280*10^∧^−7*H^∧^3−0.0117*S*H+1.9769*10^∧^−7 *S*H^∧^3)	exp(−0.3370)
D_L,CO,5s_, (mmol/min)/kPa§	Gamma	exp(0.9440+0.0205*A+0.0908*S+1.6233*10^∧^−7*H^∧^3)	exp(−2.2521)
D_L,CO,5s,hb-corr_, (mmol/min)/kPa	BCCG	exp(0.6392−0.0570*A+0.0922*S+0.0062*H+0.0005*A*H)	exp(−2.2678)
K_CO,5s_, ((mmol/min)/kPa)/L ll	Gamma	exp(0.9567+0.0576*S−0.0028*H)	exp(−2.3644)
K_CO,5s,hb-corr_, ((mmol/min)/kPa)/L	Gamma	exp(1.6187+0.0526*S−0.0092*H+8.8280*10^∧^−8*H^∧^3)	exp(−2.3626)
V_A,5s_, L†	Gamma	exp(−0.6939−0.0181*A+0.0409*S+0.0111*H+0.0003*A*H)	exp(−2.5047)
Vc, ml**	Gamma	exp(2.7298−0.0729*A−0.0268*S+0.0066*H+0.0126*A*S+0.0005*A*H)	exp(−2.1027)
Dm, (ml/min)/mmHg††	Gamma	exp(2.0825+0.0329*A+0.0573*S+0.0123*H)	exp(−1.9359)
D_L,NO_/D_L,CO,5s_	Normal	exp(0.9407+0.0458*A+0.0039*H−0.0003*A*H)	exp(−1.3398)

D_L,CO,10s_	Model	Equation( = mu)	Coefficient of variation ( = Sigma)
D_L,CO,10s_, (mmol/min)/kPa	Gamma	exp(1.0826+0.0178*A+0.0948*S+1.5419*10^∧^−7*H^∧^3)	exp(−2.1883)
D_L,CO,10s,hb-corr_, (mmol/min)/kPa	Gamma	exp(0.6371−0.0435*A+0.0939*S+0.0070*H+0.0004*A*H)	exp(−2.2356)
V_A,10s_, L	Gamma	exp(−1.2915−0.1265*A+0.0509*S+0.0213*H−2.4645*10^∧^−7 *H^∧^3+0.0009*A*H)	exp(−2.4837)
K_CO,10s_, ((mmol/min)/kPa)/L	Gamma	exp(1.2115+0.0576*S−0.0043*H)	exp(−2.2442)
K_CO,10s,hb-corr_, ((mmol/min)/kPa)/L	Gamma	exp(1.2825+0.0566*S−0.0047*H)	exp(−2.2597)

The GAMLSS model was used with a gamma distribution for all outcomes except D_L,NO_/D_L,CO,5s_ which had a normal distribution, and D_L,CO,5s,hb-corr_ that had a Box-Cox-Cole-Green distribution (BCCG). H = height in cm, A = age in years, S = sex (male = 1, female = 0),

*D_LNO_ = diffusing capacity for NO,

† V_A_ = alveolar volume,

‡ K_NO_ = D_LNO_/V_A_,

§ D_LCO_ = diffusing capacity for CO, ll K_CO_ = D_L,CO_/V_A_,

**Vc = capillary volume,

†† Dm = diffusing capacity of the alveolar membrane.

The notation _(10s)_ and _(5s)_ indicates if the outcomes were found using the D_L,CO,10s_ method or the D_L,CO,NO,5s_ method. “hb-corr” = values corrected for hemoglobin concentration.

For D_L,CO,5s_,, K_CO,5s_, D_LCO,10s_, K_CO,10s,_ and D_L,NO_/D_L,CO,5s_, reference equations were produced for both hemoglobin-corrected and non-corrected values. Vc and Dm were calculated based on the finite value of *Θ*_NO_.

Example of calculation:

The reference equation for D_L,CO,5s_ is:
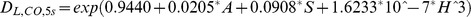
A 10-year-old boy, 140 cm tall has a predicted D_L,CO,5s_ of:
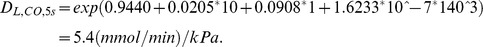
“A” is the age in years, “S” is the sex (1 for males and 0 for females), and “H” is the height in cm.

**Figure 3:**
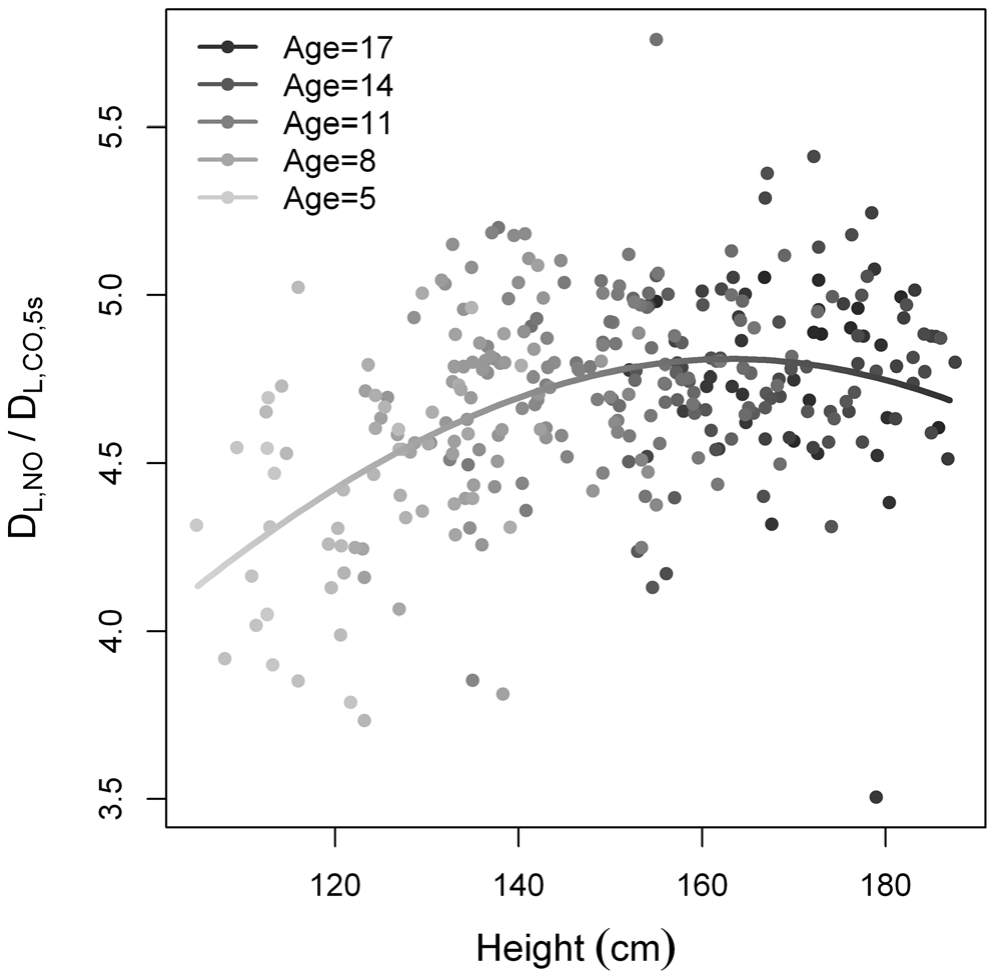
Scatter plot and curve of D_L,NO_/D_L,CO,5s_ versus height. Dot colors indicate participant age (light gray indicates the youngest and black dots the oldest).

When creating a “best-fit” model for the D_L,NO_/D_L,CO,5s_ ratio as a function of height, we saw that the ratio increased with height for the youngest participants and reached a plateau around age 14 (figure 3).

**Figure 4:**
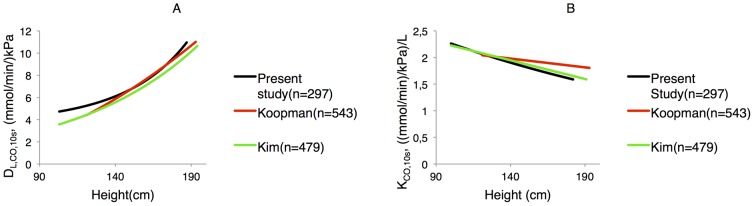
(a) D_L,CO,10s_ and (b) K_CO(10s)_ compared to recent reference equations [16], [15]. The reference equations are plotted as a function of height. All other variables were kept constant.

Our reference values for D_L,CO,10s_ and K_CO,10s_ were comparable to published reference values (figure 4a–b) [16], [19].

We have provided an Excel calculation sheet based on both GAMLSS regression and linear regression, and an example of calculation. The excel sheet is provided as **Appendix S2**.

### Quality Control

#### Repeatability of measurements in 5 to 8-year-olds and the V_IN_/FVC ratio

Young children were less likely to meet the guideline requiring less than 10% variation between two measurements of D_LCO,5s_, inspiratory volume (V_IN,5s_), D_L,CO,10s_, and V_IN,10s_. Including the mean of two measurements, not complying with ATS/ERS guidelines did not alter the reference equations (**Figure S2, Figure S3, Figure S4 and Figure S5.**).

Using the same procedure as described for the repeatability of measurements, we found little evidence that observations of V_IN_/FVC between 80% and 85% should be excluded (**Figure S6 and Figure S7**).

The influence of a given data point, such as an outlier, cannot be evaluated using residuals or Z-scores, as highly influential points will force the regression line close to it, resulting in a small residual and Z-score. We found little evidence that participants who deviated from ATS/ERS guidelines should be excluded from the estimation of reference equations for D_L,CO,5s_ and D_L,CO,10s_, as the resulting data points were not highly influential, and excluding them did not alter the Z-scores. Therefore, including them in the data analysis was acceptable.

### D_L,CO,5s_ vs. D_L,CO,10s_

**Figure 5:**
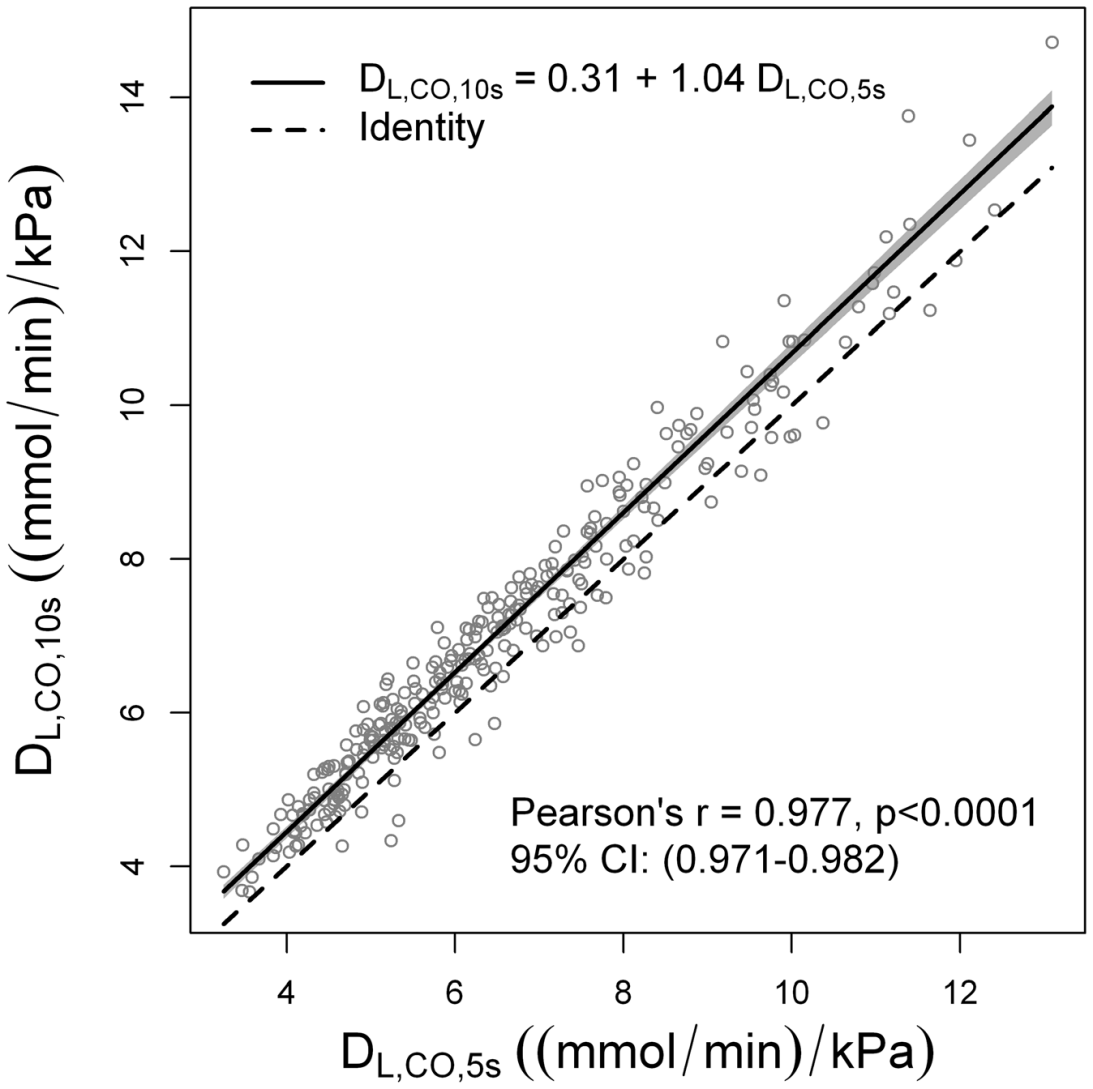
Comparison of D_L,CO,5s_ and D_L,CO,10s_. D_L,CO,5s_ and D_L,CO,10s_ were strongly correlated, with a Pearson's r = 0.977. Passing Bablok regression showed that D_L,CO,10s_ was systematically higher by a constant of 0.31, and proportionally higher by a factor of 1.04.

D_L,CO,10s_ was significantly higher than D_L,CO,5s_ (paired t-test p<0.0001) but as expected, D_L,CO,10s_ and D_L,CO,5s_ were strongly correlated (r = 0.98, p<0.0001). Similarly, using the Passing Bablok regression, we found a systematic difference, as well as a proportional difference (figure 5).

**Figure 6:**
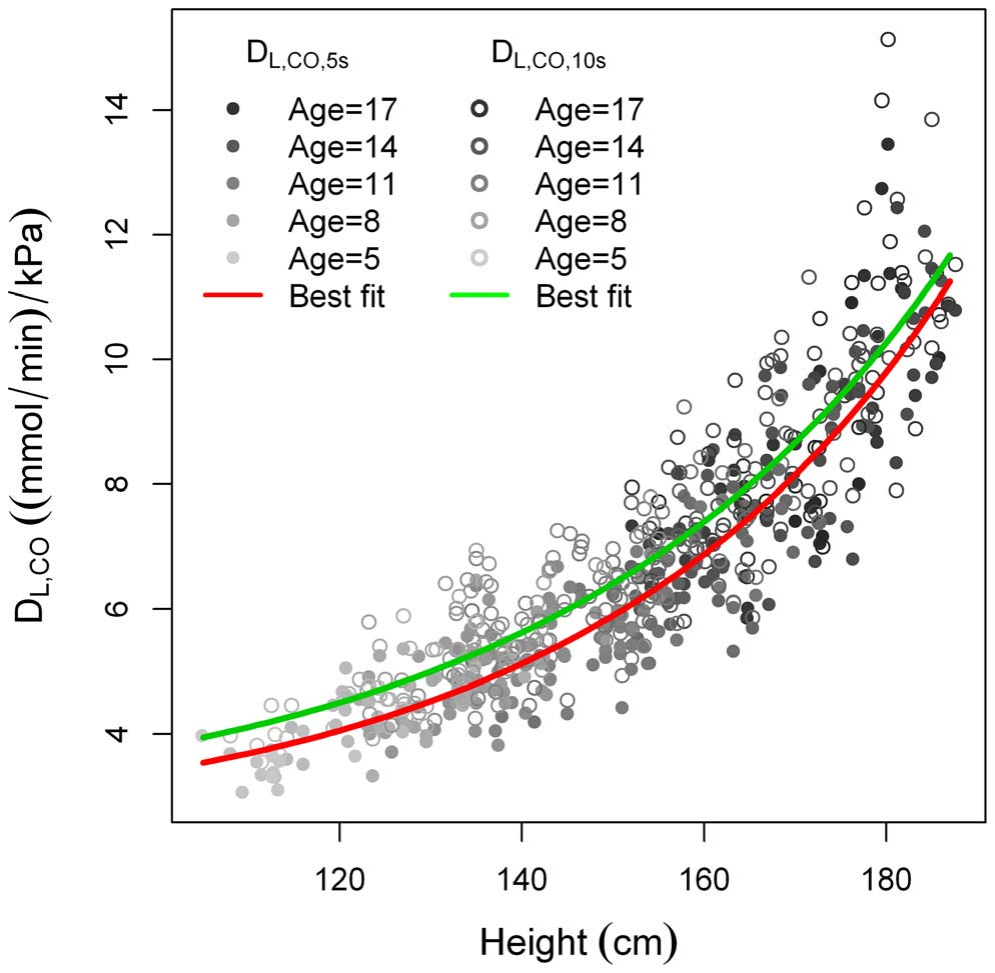
D_L,CO,5s_ and D_L,CO,10s_ plotted as a function of height.

**Figure 7:**
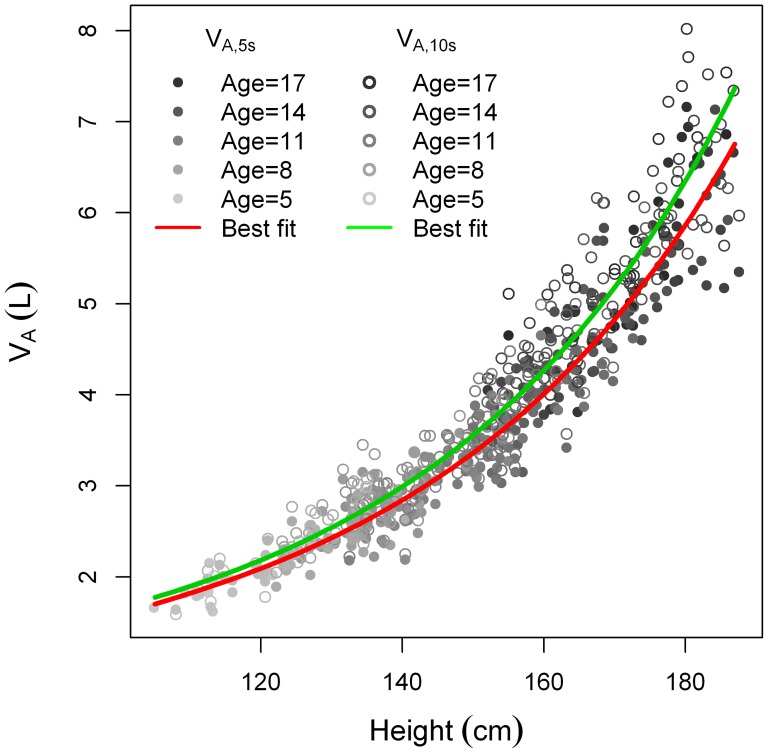
Va_5s_ and Va_10s_ plotted as a function of height.

When plotting D_L,CO,10s_ and D_L,CO,5s_ as a function of height, we found D_L,CO,10s_>D_L,CO,5s_. (Figure 6) as well as V_A,10s_>V_A,5s_ (Figure 7).

### Vc and Dm

**Figure 8:**
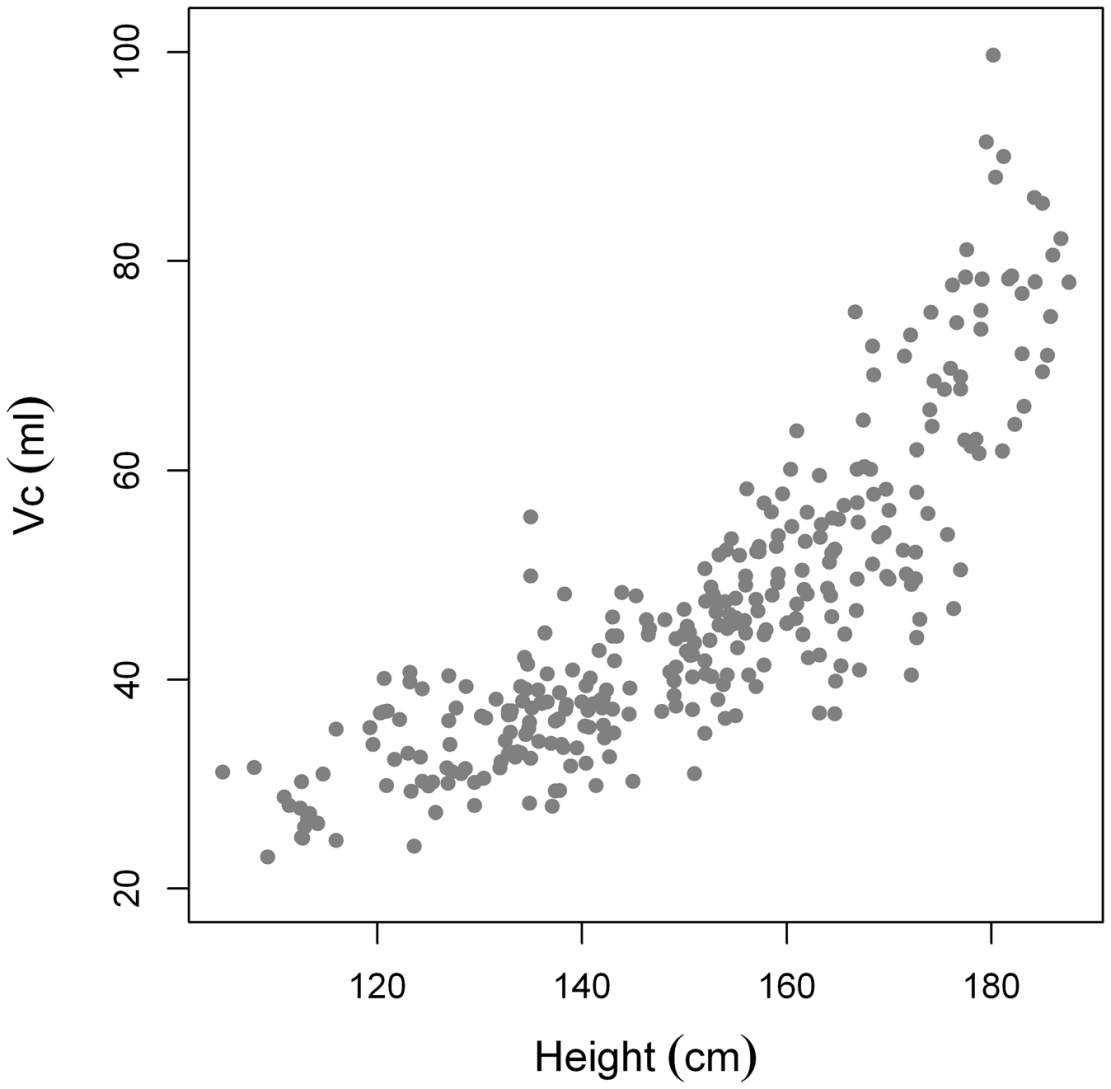
Vc plotted as a function of height.

**Figure 9:**
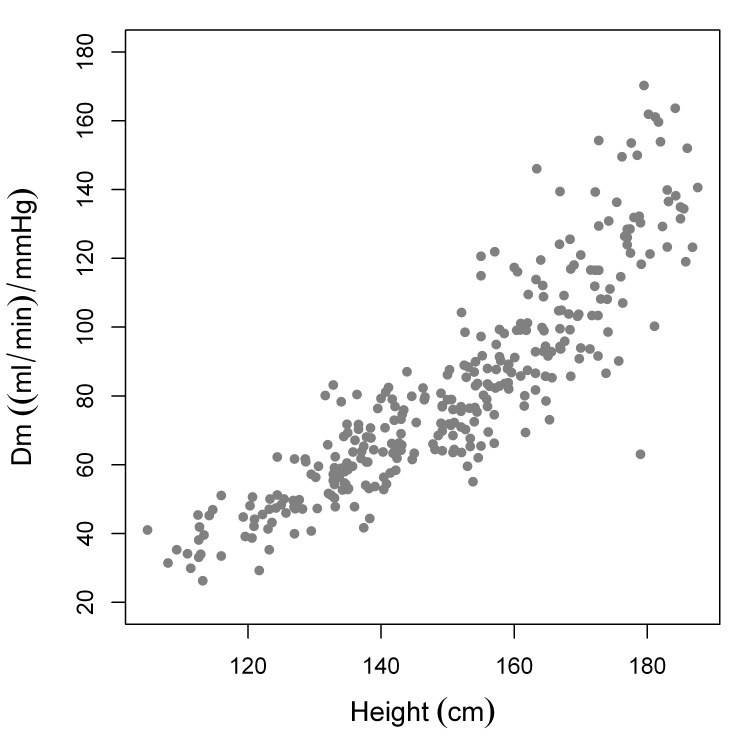
Dm plotted as a function of height.

Vc and Dm both increase with height. (Figure 8 and 9).

## Discussion

This is the first study to establish reference equations for the outcomes of D_L,CO,NO,5s_, including the calculated outcomes: Vc, Dm, and the D_L,NO_/D_L,CO,5s_ ratio, in a large group of healthy children of European descent. The measurement and evaluation of Vc and Dm can potentially provide valuable information about the causes of decreased diffusing capacity and the development and progression of lung disease or vascular disorders from the age of 5 years.

Vc and Dm are not entirely accepted as robust parameters, partially due to the lack of reference equations, which limits their clinical and scientific use. A more problematic issue is the current lack of agreement regarding the true value of *Θ*CO and the relationship with arterial oxygen pressure. The calculated Vc is dependent on this value and will vary depending on which equation is used. The equation utilized in this paper was based on measurements performed at pH 7.4 [24], for conventional reasons, and because it is closer to a physiological value. Finally, another topic of debate is α, the ratio of NO to CO diffusivity. In the present study, a physical α value of 1.97 was used [4], but an alternative empiric value of 2.42 has been proposed [4], [32].

The D_L,NO_/D_L,CO,5s_ ratio has been proposed as a measure of the relative properties of Dm and Vc [33]. Previous studies have concluded that the D_L,NO_/D_L,CO,5s_ ratio in adults is independent of age [12], [34]. Figure 3 is produced via a “best fit”-model for the available data, and may not reflect the true bio-physical relationship between height and this ratio. That being said, we found that the ratio increased with height until mid pubertal age at approximately 14 years and then reached a plateau.

We have shown that both Vc and Dm increase with height(Figure 8 and 9). As stated in the introduction, according to current opinion the diffusing capacity of NO (D_L,NO_) reflects both Dm and *Θ*_CO_ 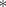
Vc, whereas D_L,CO_ primarily reflects Vc. With increasing height D_L,NO_ will increase relatively more than D_L,CO_ leading to the D_L,NO_/D_L,CO,5s_ reaching a plateau around 140 cm.

The lower D_L,NO_/D_L,CO,5s_ in younger and smaller children may be due to a greater rate of capillary growth compared to lung surface growth or to a relatively thicker membrane in the young. As height increases with age, a compensatory relatively larger increase in Dm would result in an increasing ratio. Alveolarization has been shown to continue through out childhood and adolescence [35] and could help explain the increase in Dm. The literature on this topic is scarce, and future studies are needed to understand and interpret the effect of age and height on the D_L,NO_/D_L,CO,5s_ ratio.

### D_L,CO,10s_

The reference values calculated in the present study for D_L,CO,10s_ were slightly higher than existing, published reference values. One possible reason for this difference is that the present study population included children with both parents of European descent, whereas Koopman et al. included children with only one parent of European descent [16]. Ethnic differences in D_L_ in adults are small, but well established [36], [37]. Another reason for the difference is the pulmonary function equipment; the equipment used in the present study and by Koopman et al. were very similar, whereas the apparatus' used by Kim et al. [19] at their two locations were from two different manufacturers. Furthermore, even with the same apparatus, differences in software including various corrections, may lead to the observed differences.

Our results stress the importance of creating reference equations specific for a single population, or at least validating existing reference equations prior to implementing them in a laboratory setting.

### D_L,CO,5s_ *vs.* D_L,CO,10s_

Although the primary purpose of measuring D_L,CO,10s_ was to secure a meaningful correlation to the much more scarcely described D_L,CO,NO,5s_ technique, we secondarily wished to compare D_L,CO_ measured by the two techniques. As expected we found a significant, systematic difference between D_L,CO,5s_ and D_L,CO,10s_. The difference in D_L,CO_ can be caused by a number of factors, as the two methods vary in a number of ways. See table 1. First, methane and helium may have different distributions in the lung owing to their respective physical properties; they have also different solubility in tissue. This may lead to a difference in V_A_ and a resulting difference in D_L,CO_ as D_LCO_ = K_CO_ 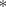
V_A_. Second, the sample method varies, with a physical gas sample being collected in the case of D_L,CO,NO,5s_, whereas a virtual sample was constructed from flow and gas concentration signals in the case of D_L,CO,10s_. Finally, we speculate if the difference in the kinetics of NO and CO in binding with hemoglobin may play a roll.

Older studies on D_L,CO,10s_ focusing on varying breath-hold times, keeping all other factors constant, have shown that breath-hold time alone, influences K_CO_, leading to a decreased D_L,CO_ with an increased breath-hold time [38]. This is in contrast to our findings, but apparently the mentioned differences in methodology other than breath-hold, have a greater impact on D_L,CO_.

In summary, the two methods vary in a number of ways and D_L,CO_ measured using D_L,CO,NO,5s_ and D_L,CO,10s_ cannot be used interchangeably for monitoring pulmonary disease. More research is required to determine how the mentioned factors combine to influence D_L,CO_. A given value of D_L,CO_ can only be evaluated using reference equations produced with the same methodology and breath-hold time as recently confirmed [39].

### CO and NO backpressure

The participants performed two or three tests, and rarely up to six repetitions of both D_L,CO,NO,5s_ and D_L,CO,10s_, resulting in a maximum of 12 tests in a single sitting. Repeating measurements of D_L,CO,10s_ leads to an accumulation of CO in the blood, creating CO backpressure and decreasing D_L,CO_. However, recent work by Zavorsky showed that up to 12 tests can be performed in adults without significantly lowering the D_L,CO_. Furthermore, in regards to D_L,CO,NO,5s_, up to 22 repetitions does not lead to a decrease in D_L,NO_ [40]. Taking this into account, we have no reason to suspect CO or NO backpressure to be of influence in the present study.

### Quality control

Measuring lung function in this age group requires extra time and effort, but it is feasible. Most of the young children were able to perform the measurements according to ATS/ERS guidelines, but some had greater variability between measurements than normally accepted. This difference was partially due to the limited attention span of the children, who were not always able to perform repeated tests if the first two measurements did not comply with the ATS/ERS standard of a maximum 10% difference between measurements. We included measurements with greater variability, as they did not affect the estimated reference equations. Accepting greater variation in children makes sense if the alternative is to discard measurements completely.

The ATS/ERS guidelines recommend an acceptance criterion of V_IN_/VC ≧85% for adults. The recommendation is based on D_LCO10s_ measured in a large group of adults, where 72%, 86%, and 92% of the participants were able to achieve a V_IN_/VC ratio of 90%, 85%, and 80%, respectively. Therefore, the recommended ratio, i.e., 85%, is a relatively arbitrary value and the guidelines state that V_IN_/VC <85% may still have clinical utility [2].

Although most of our participants were able to inhale to more than 85% of FVC, some were not, despite multiple attempts and prompting and otherwise performing an adequate maneuver.

We found no differences between reference equations including measurements with V_IN_/FVC >80% and reference equations only including V_IN_/FVC >85%.

In summary, we accepted measurements that did not meet ATS/ERS quality criteria because these measurements had no effect on the resulting equations. In the future, specific pediatric guidelines for both D_L,CO,NO,5s_ and D_L,CO,10s_ would be relevant.

### Strengths and limitations

The primary strength of this study is the large and acceptable age distribution of healthy children and adolescents from varying demographic backgrounds. Furthermore, this study was completed in two laboratory setups with identical equipment, as described in the online supplement. The same two technicians performed all measurements, resulting in a high level of repeatability and a systematic approach. In addition, we included children as young as 5 years of age, expanding our ability to adequately evaluate advanced pulmonary function in this age group. Finally, our calculated reference equations for D_L,CO,10s_ corresponded well to recently published equations, in particular those of Koopmans et al. [16]

In hindsight, it would have been beneficial to include a “young adult” group, 18–22 years old, in this study, as it would open up the possibility of bridging reference equations to include children, adolescents, young adults, and adults.

For the youngest children with a VC<1.5 liters, we reduced the discard volume to 500 ml. If the VC is even lower, as in the case of disease, this method may not be suitable. Multiple other techniques exist for D_L,CO_. These include the steady state method, particularly suitable for infants or anaesthetized patients, or the rebreathing and intrabreath method, that both require cooperation, but can be performed in patients with lower lung volumes [41]. So far these modifications have not been applied to D_L,CO,NO_.

### Conclusion

This study is the first to create pediatric reference equations for the outcomes D_L,CO,5s_, D_L,NO_, and the calculated outcomes D_L,NO_/D_L,CO,5s_, Vc, and Dm measured by D_L,CO,NO,5s_ in healthy children and adults, of European descent. These equations are based on a large population with a broad age range, including children as young as 5 years of age. We expect that the present reference equations can be applied to similar populations throughout Europe, Australia and North America.

We hope that having reliable reference equations for Dm, Vc, and D_L,NO_/D_L,CO,5s_ will lead to improved diagnostic evaluation and provide a monitoring tool for the treatment of children presenting with diffuse interstitial lung disease, whether it is a pure alveolocapillary membrane disturbance or pulmonary micro vascular disease. In particular, we believe that the D_L,NO_/D_L,CO,5s_ ratio has great potential, as it is independent of the assumptions and models used to calculate Vc and Dm, that may be easily questionable. However, the clinical utility of Vc, Dm, and D_L,NO_/D_L,CO,5s_ still needs to be evaluated in future studies. We acknowledge that multicenter studies are required for external validation of these results. We invite researchers to compare their results, in children with well known pathological features of the lung, with the results of this study. This will achieve increased understanding of the physiological meaning of the described measurements and their application in the early detection and monitoring of diseases.

## Supporting Information

Figure S1 **Age and gender distribution of participants.**
(TIFF)

Figure S2 **Quality control.** Participants with more than 10% difference between two independent measurements of D_L,CO,5s_ were evaluated, as this is in contrast to ATS/ERS guidelines.
(TIFF)

Figure S3 **Quality control.** Participants with more than 10% difference between two independent measurements of inspiratory volume (V_IN,5s_) were evaluated, as this is in contrast to ATS/ERS guidelines.
(TIFF)

Figure S4 **Quality control.** Participants with more than 10% difference between two independent measurements of D_L,CO,10s_ were evaluated, as this is in contrast to ATS/ERS guidelines.
(TIFF)

Figure S5 **Quality control.** Participants with more than 10% difference between two independent measurements of inspiratory volume V_IN,10s_ were evaluated, as this is in contrast to ATS/ERS guidelines.
(TIFF)

Figure S6 **Quality control.** Measurements of V_IN,5s_/FVC between 80% and 85% were evaluated as ATS/ERS requires values >85%.
(TIFF)

Figure S7 **Quality control.** Measurements of V_IN,10s_/FVC between 80% and 85% were evaluated as ATS/ERS requires values >85%.
(TIFF)

Appendix S1 **Age distribution.**
(DOCX)

Appendix S2 **Excel worksheet.**
(XLSX)
