# Correction: The Single-Breath Diffusing Capacity of CO and NO in Healthy Children of European Descent

**DOI:** 10.1371/journal.pone.0179097

**Published:** 2017-06-01

**Authors:** Astrid Thomas, Birgitte Hanel, Jacob L. Marott, Frederik Buchvald, Jann Mortensen, Kim G. Nielsen

The Data Availability statement for this paper is incorrect. The correct statement is: Anonymized data are available from the figshare repository: https://doi.org/10.6084/m9.figshare.5012072.
