# Supplementary figures and images for: The Single-Breath Diffusing Capacity of CO and NO in Healthy Children of European Descent
Source: PLoS One. 2014 Dec 16;9(12):e113177. doi: 10.1371/journal.pone.0113177 (PMC4267784; doi:10.1371/journal.pone.0113177)

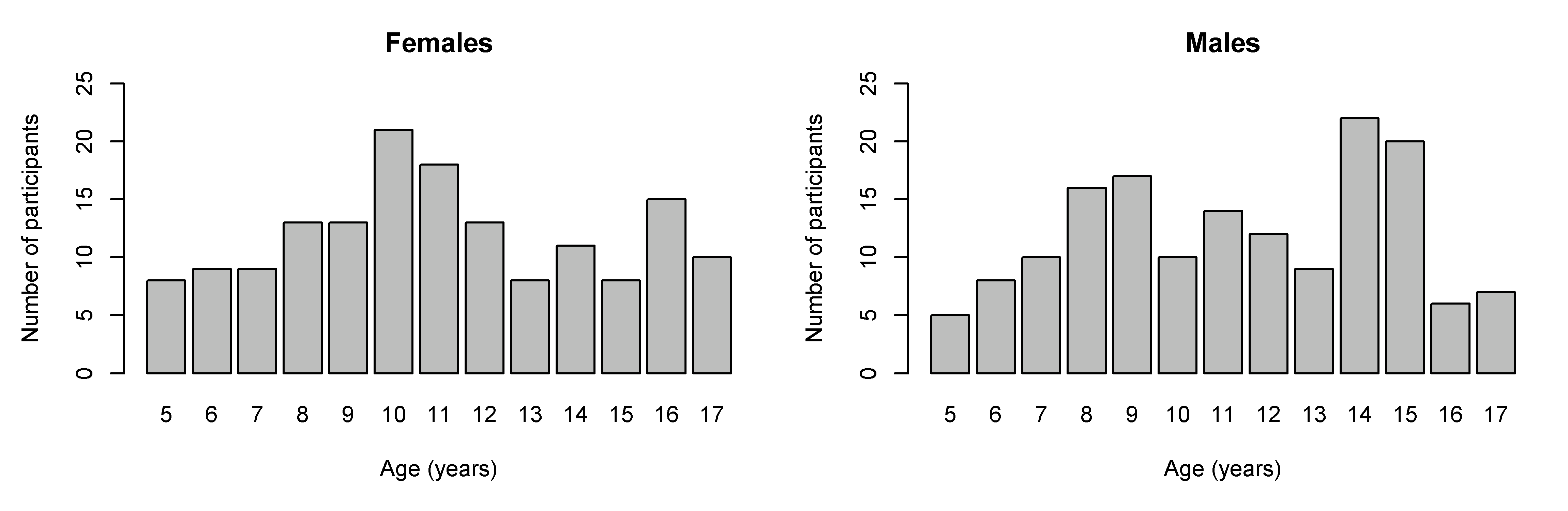

Supplement: Figure S1 — Age and gender distribution of participants. (TIFF) [file pone.0113177.s001.tiff]

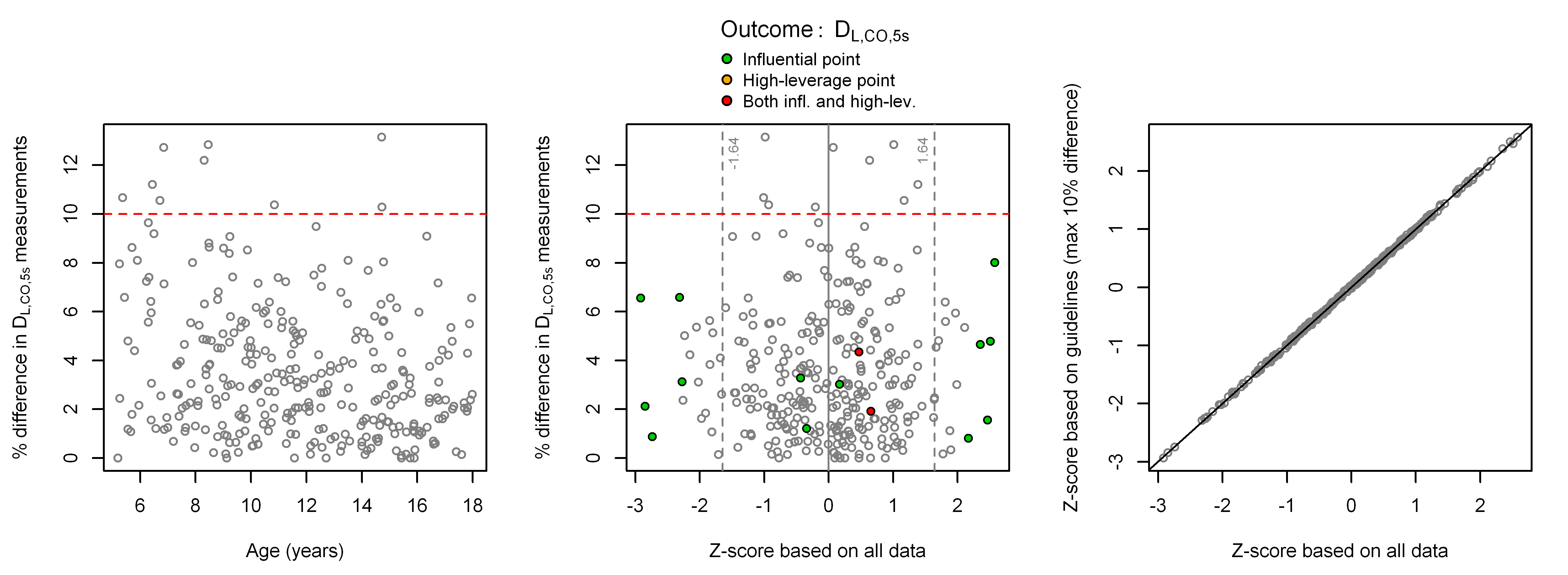

Supplement: Figure S2 — Quality control. Participants with more than 10% difference between two independent measurements of DL,CO,5s were evaluated, as this is in contrast to ATS/ERS guidelines. (TIFF) [file pone.0113177.s002.tiff]

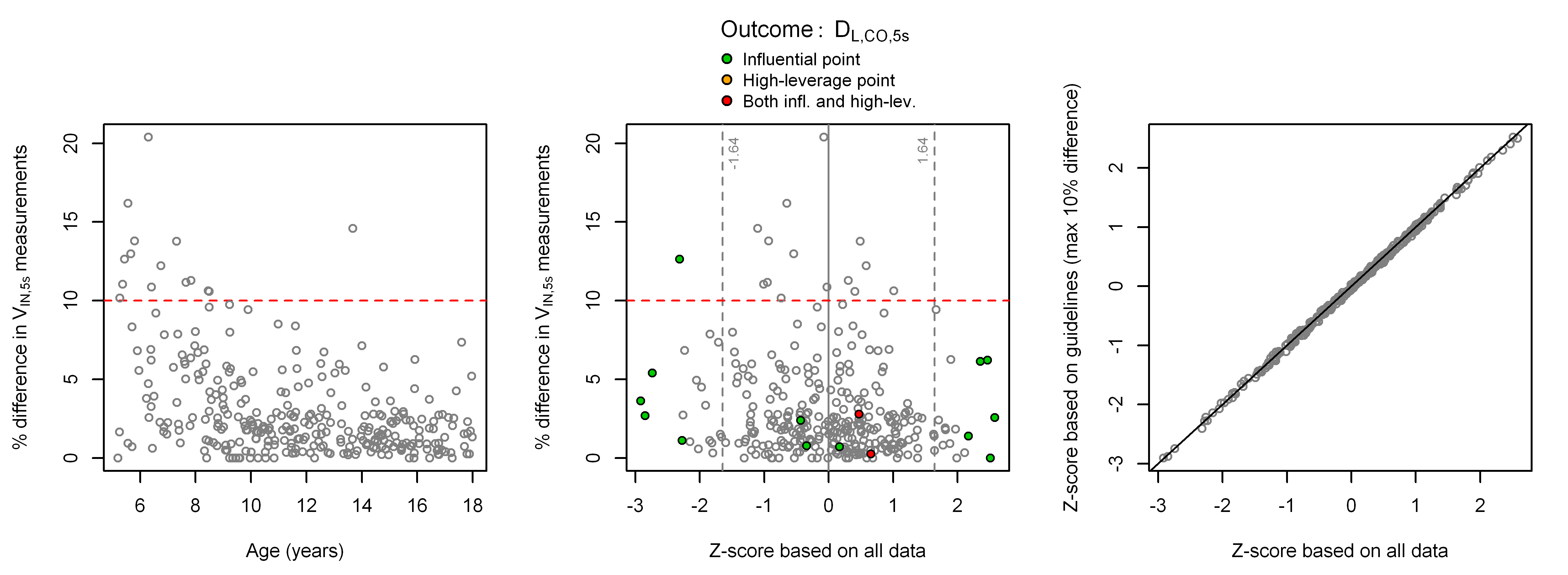

Supplement: Figure S3 — Quality control. Participants with more than 10% difference between two independent measurements of inspiratory volume (VIN,5s) were evaluated, as this is in contrast to ATS/ERS guidelines. (TIFF) [file pone.0113177.s003.tiff]

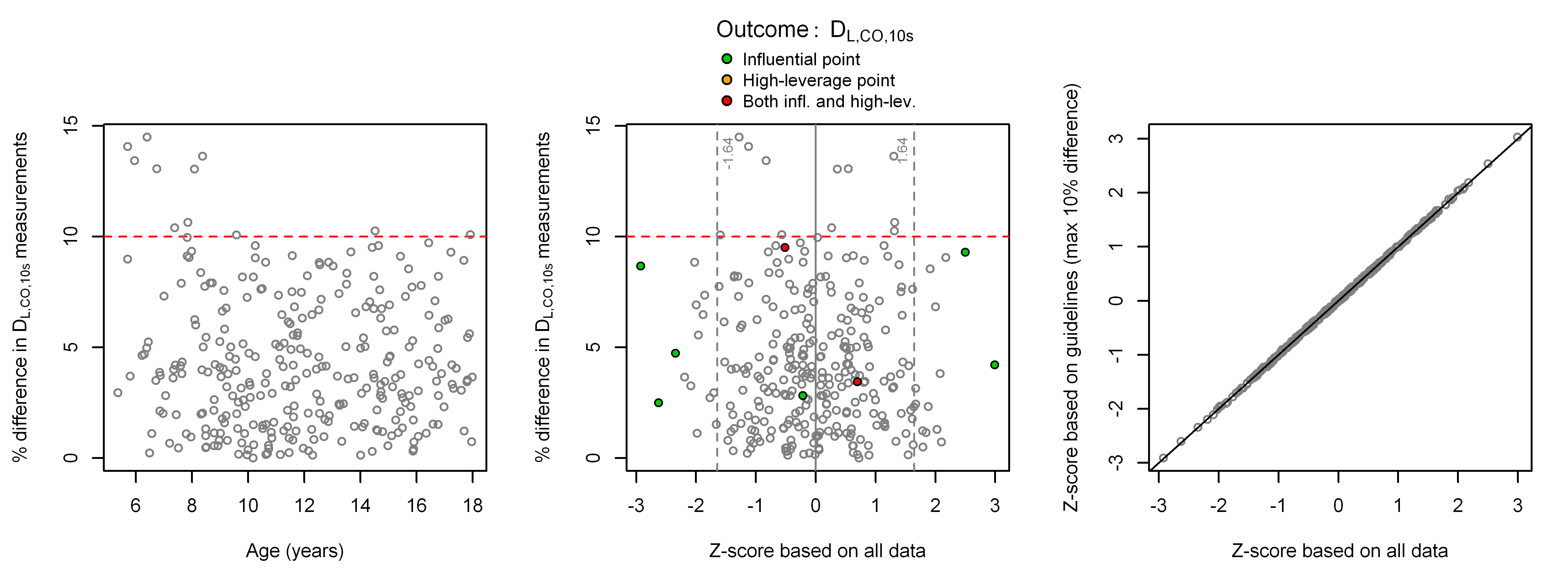

Supplement: Figure S4 — Quality control. Participants with more than 10% difference between two independent measurements of DL,CO,10s were evaluated, as this is in contrast to ATS/ERS guidelines. (TIFF) [file pone.0113177.s004.tiff]

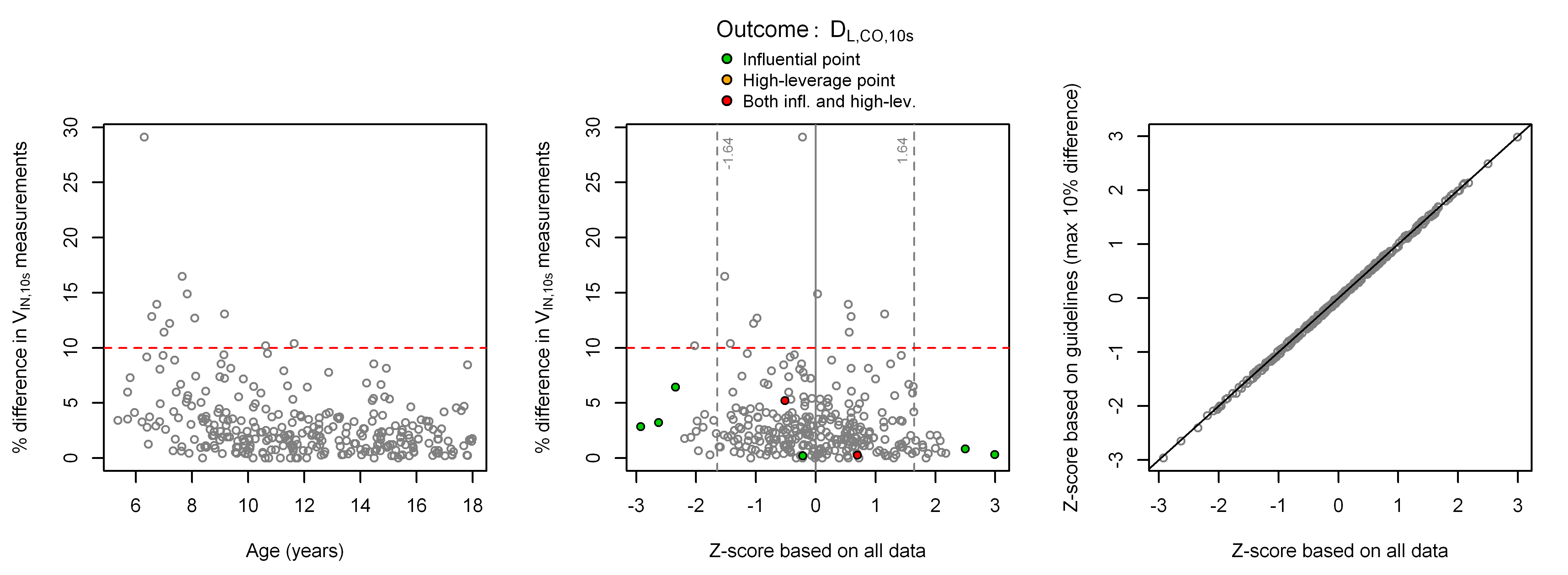

Supplement: Figure S5 — Quality control. Participants with more than 10% difference between two independent measurements of inspiratory volume VIN,10s were evaluated, as this is in contrast to ATS/ERS guidelines. (TIFF) [file pone.0113177.s005.tiff]

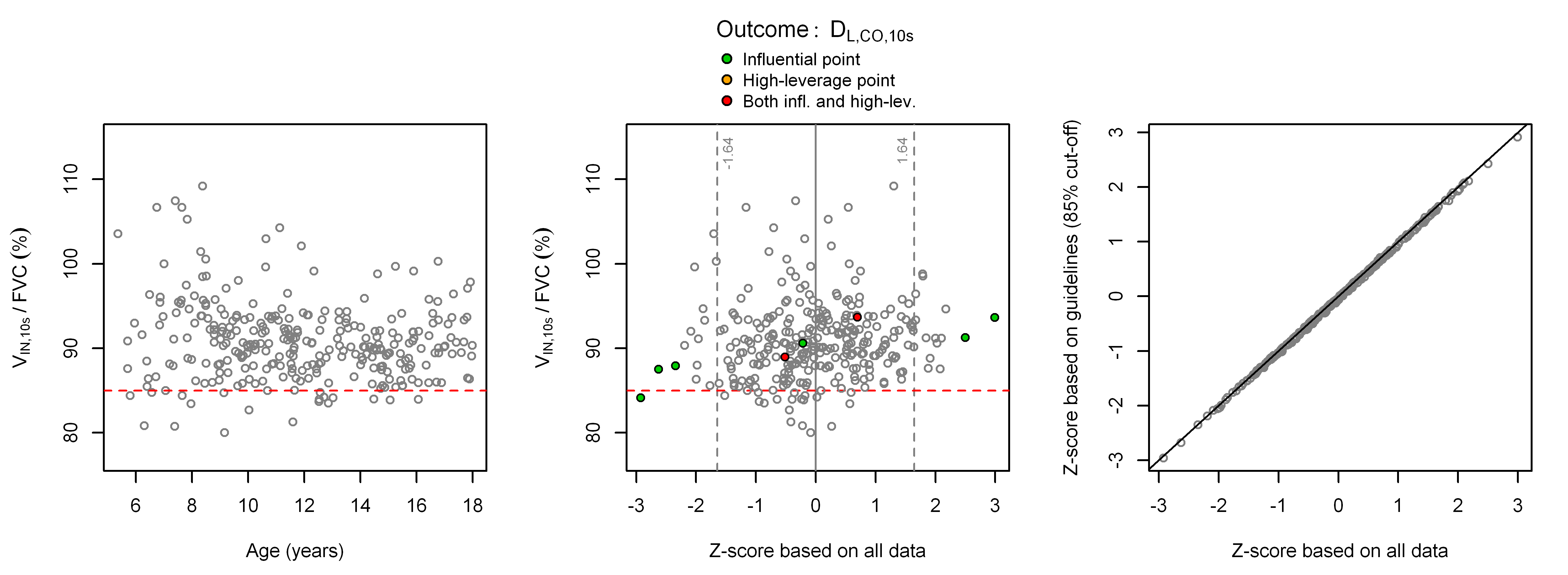

Supplement: Figure S6 — Quality control. Measurements of VIN,5s/FVC between 80% and 85% were evaluated as ATS/ERS requires values >85%. (TIFF) [file pone.0113177.s006.tiff]

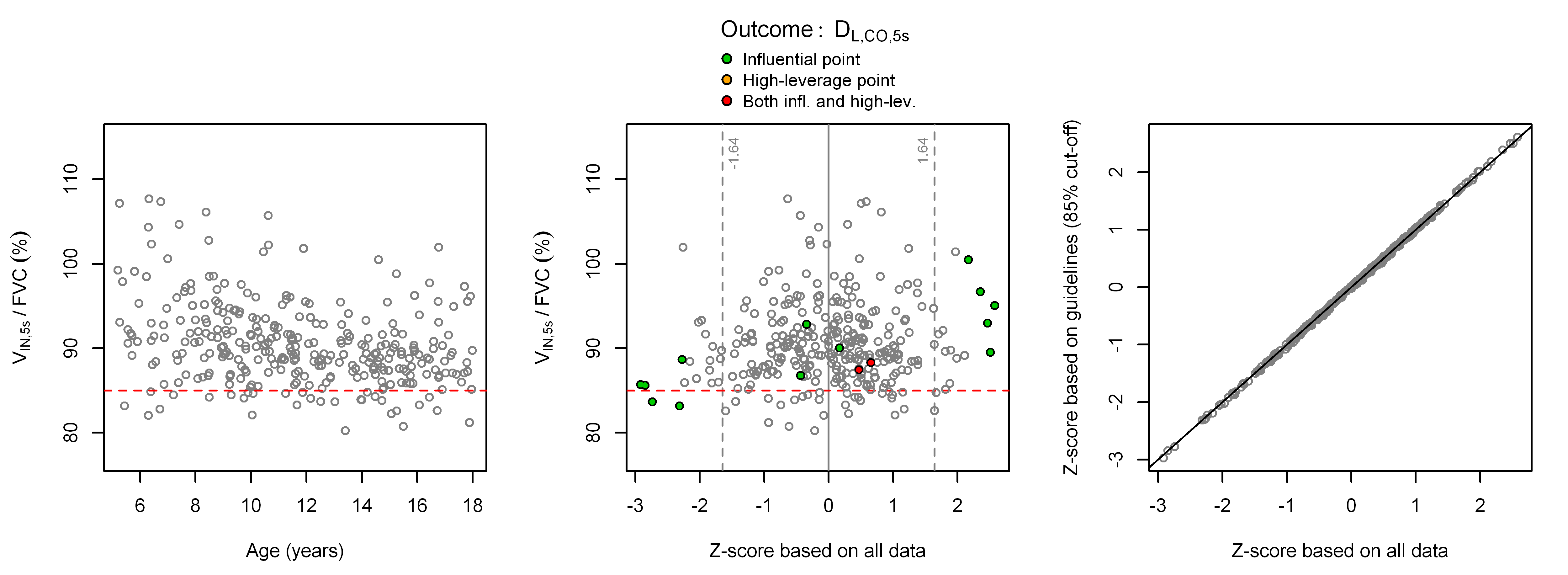

Supplement: Figure S7 — Quality control. Measurements of VIN,10s/FVC between 80% and 85% were evaluated as ATS/ERS requires values >85%. (TIFF) [file pone.0113177.s007.tiff]
