# Supplementary material for: The Single-Breath Diffusing Capacity of CO and NO in Healthy Children of European Descent
Source: PLoS One. 2014 Dec 16;9(12):e113177. doi: 10.1371/journal.pone.0113177 (PMC4267784; doi:10.1371/journal.pone.0113177)
Supplement: Appendix S1 — Age distribution. (DOCX) [file pone.0113177.s008.docx]

**Qulity Control report on the two sets of equipment using the Jaeger-JQM syringe (3 Liter) DL,CO test.**

| **JQM_SYRINGE_x_LAB_x FROM FACTORY IN SYSTEM USED AT SCHOOLS at two points** | | | | | |
| --- | --- | --- | --- | --- | --- |
| Channel | Unit | Low | Measured | High | Pass/Fail |
| 1 VA(CH4) | Liter | 2.8500 | 3.1300 | 3.1500 | Pass |
| 1 VA(CO) | Liter | 2.8500 | 3.0300 | 3.1500 | Pass |
| 1 VALIN | % | 96.0 | 103.1 | 104.0 | Pass |
| 2 VA(CH4) | Liter | 2.910 | 3.040 | 3.0800 | Pass |
| 2 VA(CO) | Liter | 2.910 | 2.980 | 3.0800 | Pass |
| 2 VALIN | % | 96.0 | 102.1 | 104.0 | Pass |

| **JQM_SYRINGE_x_LAB_x SYSTEM USED AT SCHOOLS, AFTER STUDY, at two points** | | | | | |
| --- | --- | --- | --- | --- | --- |
| Channel | Unit | Low | Measured | High | Pass/Fail |
| 1 VA(CH4) | Liter | 2.8500 | 3.0200 | 3.1500 | Pass |
| 1 VA(CO) | Liter | 2.8500 | 3.0200 | 3.1500 | Pass |
| 1 VALIN | % | 96.0 | 104.0 | 104.0 | Pass |
| 2 VA(CH4) | Liter | 2.910 | 3.000 | 3.0800 | Pass |
| 2 VA(CO) | Liter | 2.910 | 2.970 | 3.0800 | Pass |
| 2 VALIN | % | 96.0 | 100.9 | 104.0 | Pass |

| **JQM_SYRINGE_x_LAB_x FROM FACTORY IN SYSTEM USED AT HOSPITAL at two points** | | | | | |
| --- | --- | --- | --- | --- | --- |
| Channel | Unit | Low | Measured | High | Pass/Fail |
| 1 VA(CH4) | Liter | 2.8500 | 3.060 | 3.1500 | Pass |
| 1 VA(CO) | Liter | 2.8500 | 3.0400 | 3.1500 | Pass |
| 1 VALIN | % | 96.0 | 101.1 | 104.0 | Pass |
| 2 VA(CH4) | Liter | 2.910 | 2.990 | 3.0800 | Pass |
| 2 VA(CO) | Liter | 2.910 | 2.970 | 3.0800 | Pass |
| 2 VALIN | % | 96.0 | 101.5 | 104.0 | Pass |

| **JQM_SYRINGE_x_LAB_x SYSTEM USED AT HOSITAL AFTER STUDY, at two points** | | | | | |
| --- | --- | --- | --- | --- | --- |
| Channel | Unit | Low | Measured | High | Pass/Fail |
| 1 VA(CH4) | Liter | 2.8500 | 3.0100 | 3.1500 | Pass |
| 1 VA(CO) | Liter | 2.8500 | 2.9200 | 3.1500 | Pass |
| 1 VALIN | % | 96.0 | 99.9 | 104.0 | Pass |
| 2 VA(CH4) | Liter | 2.910 | 3.020 | 3.0800 | Pass |
| 2 VA(CO) | Liter | 2.910 | 2.990 | 3.0800 | Pass |
| 2 VALIN | % | 96.0 | 101.2 | 104.0 | Pass |
